# Relevance and clinicopathologic relationship of BRAF V600E, TERT and NRAS mutations for papillary thyroid carcinoma patients in Northwest China

**DOI:** 10.1186/s13000-019-0849-6

**Published:** 2019-07-12

**Authors:** Meiling Huang, Changjiao Yan, Jingjing Xiao, Ting Wang, Rui Ling

**Affiliations:** Department of Thyroid, Breast, and Vascular Surgery, Xijing Hospital, The Fourth Military Medical University, Xi’an, 710032 China

**Keywords:** BRAF V600E mutation, TERT mutation, NRAS mutation, Co-mutations, Papillary thyroid carcinoma

## Abstract

**Background:**

To determine the relevance of the single or combination mutations of BRAF V600E, TERT, and NRAS genes and the clinicopathologic relationship in papillary thyroid cancer (PTC).

**Methods:**

Patients with PTC were enrolled into the study between February 2018 and April 2019. Based on the number of mutant genes, we classified the participants into single BRAF V600E mutation group, double mutations group and no mutation group. Single factor and multiple logistic regression analyses were applied to explore the independent factors. Review Manager 5.3 was used for meta-analysis to review the clinical efficacy of gene co-mutations.

**Results:**

Finally, 483 patients were enrolled into the study and 419 (86.7%) of them harbored BRAF V600E mutation. TERT or NRAS mutation was likely to coexist with BRAF V600E mutation in PTC. BRAF V600E and NRAS promoter co-mutations was identified in 6 patients, with a prevalence of 1.2%. Prevalence of BRAF V600E and TERT coexistence in PTC was 2.1%. Significant differences were found among age, pathology, multifocality, bilateral lesions, lymph node metastasis, and 131I radiotherapy, *P* < 0.01. Multiple logistic regression analyses demonstrated that age [odds ratio (OR) = 1.044, 95% *confidence interval (CI)* = 1.013–1.076; *P* = 0.006], lymph node metastasis [OR = 0.094, 95% *CI* = 0.034–0.264; *P* < 0.001], 131I radiotherapy [OR = 7.628, 95% *CI* = 2.721–21.378; *P* < 0.001] were risk factors for BRAF V600E mutation. Besides, age [OR = 1.135, 95% *CI* = 1.069–1.205; *P* < 0.001], multiple leisions [OR = 4.128, 95% *CI* = 1.026–16.614; *P* = 0.046], pathology [OR = 3.954, 95% *CI* = 1.235–12.654; *P* = 0.021] were independent factors for combination mutations. Meta-analysis showed significant association of BRAF V600E+/TERT+ co-mutations with lymph node metastasis, multifocality, distant metastasis, tumor recurrence, extrathyroidal extension, and dead of disease.

**Conclusions:**

Prevalence of BRAF V600E mutation in Northwest China was higher than other areas. Age, multiple lesions, and pathology were independent factors for double mutation of BRAF V600E/TERT or BRAF V600E/NRAS. Coexistence of BRAF V600E and TERT promoter mutations was significantly correlated with poor outcome.

## Introduction

Thyroid cancer is the most common endocrine malignancy, and its global incidence has rapidly increased in recent decades [1]. Papillary thyroid carcinoma (PTC), which is derived from the follicular epithelium, represents 80 to 85% of thyroid malignancies. Although PTC is highly curable in general, approximately 10% of patients are destined as progressive disease [2]. Thus, the molecular-based risk stratification has been emphasized to compare treatment-associated benefits. Recently, improved understanding of the molecular pathogenesis and the identification of molecular markers are of high clinical significance, indicating the diagnosis and prognosis of PTC.

Molecular markers have been focused so far, such as BRAF V600E, telomerase reverse transcriptase (TERT) and NRAS, which might be potential prognostic factors for FTC. BRAF V600E mutation was correlated with more aggressive and iodine-resistant phenotypes, providing valuable prognostic information for thyroid cancer [3]. Similarly, TERT promoter mutation was associated with aggressive thyroid tumor characteristics, tumor recurrence, and patient mortality [4]. NRAS gene, the most frequent mutant gene of the RAS gene family, was related to increased risk of distant metastasis [5, 6]. However, features of gene mutation from different regions are different. In Australian urban population, 68% of PTC patients were identified with BRAF V600E mutation [7]. In Middle Eastern, TERT promoter mutation was harbored in 6.5% PTC patients [8]. For PTC patients from Greek, low prevalence of TERT promoter (3.4%), BRAF V600E (17%), and RAS mutations (3.4%) was detected [9]. In China, data of gene mutation for PTC was relatively limited. In 2018, Liang J et al. reported 72.4% of BRAF V600E mutation and 2.8% of RAS mutation among 355 Chinese PTC patients [10]. In China, it is essential to achieve more evidence of genetic events as trustworthy prognostic markers for risk stratification and patient management.

Considering the synergistic effects of mutant genes, coexistence of gene mutation should be emphasized. BRAF V600E promoter mutation, in combination with TERT or RAS mutation, was recognized as clinically important diagnostic and prognostic genetic markers for thyroid cancer. TERT, a predominant determinant for controlling the activity of telomerase, was likely to coexist with BRAF V600E mutation in thyroid cancer [11]. In 2016, Sun J et al. found that 94.7% PTC patients with TERT promoter mutation were detected with BRAF V600E mutation [12]. In 2017, Vuong HG et al. claimed that the combination of BRAF V600E and TERT promoter mutations indicated increasing risk of aggressiveness of PTC than TERT or BRAF V600E mutation alone [13]. In this study, we focused on the prevalence of BRAF V600E, TERT and NRAS mutations and their association with clinicopathological features in PTC patients from Northwest China.

## Materials and methods

### Participants

This retrospective study included 483 patients (127 men and 356 women) admitted to Xijing Hospital, between February 2018 and April 2019. The fundamental features were shown in Table 1. All patients underwent preoperative ultrasound and fine-needle aspiration biopsy tests. Total or near-total thyroidectomy, cervical lymph node dissection, and radioiodine therapy were pursued as clinically determined. Pathological diagnosis was established following the World Health Organization criteria and confirmed by expert thyroid cancer pathologists. All patients provided written informed consent. Ethical approval for the study was provided by the Ethical Committees of Xijing Hospital.

**Table 1:** Baseline characteristics

Index	Data (*N* = 483)
Sex	
Male	127 (26.3%)
Female	356 (73.7%)
Age	
Average age	43.15 ± 11.25
Median age	43 (14–79)
Pathology	
PTC	187 (38.7%)
PTMC	296 (61.3%)
Lesion number	
Single lesion	342 (70.8%)
Multiple lesions	141 (29.2%)
Lesion location	
Unilateral	404 (83.6%)
Bilateral	79 (16.4%)
Gene mutation	
BRAF V600E mutation alone	419 (86.7%)
BRAF V600E/TERT co-mutation	10 (2.1%)
BRAF V600E/NRAS co-mutation	6 (1.2%)
No mutations in BRAF V600E/TERT/NRAS	48 (9.9%)

### Genomic DNA isolation

Formalin-fixed and paraffin-embedded (FFPE) tumor tissue was achieved for human genomic DNA isolation, using the AmoyDx**®** FFPE DNA Kit (Amoy Diagnostics Co., Ltd., Xiamen, China). Selection of the most representative areas was made by an experienced thyroid pathologist. Before DNA isolation, paraffin was removed by xylene-ethanol extraction, and lysed overnight with 20 μL proteinase K in a 56 °C rotating incubator. DNA purification was performed using the QIAamp DNA Mini Kit (Qiagen GmBH, Hilden, Germany), according to the manufacturer’s instructions. The yielded DNA with sufficient quantity and quality was stored at − 40 °C.

### Detection of the BRAF V600E mutation

BRAF V600E mutation was determined by polymerase chain reaction (PCR) assay. The gene was performed in a final volume of 50 μl using as template 100–300 ng of genomic DNA, with 1× buffer including 1.5 mM MgCl_2_, 0.2 mM dNTPs, 25 pmoles of each (Forward, Reverse) primer and 1 unit of Taq polymerase (Kapa Biosystems). PCR was run with a step-down protocol: 95 °C for 5 min × 1 cycle, 95 °C for 25 s, 64 °C for 20 s, and 72 °C for 20 s × 15 cycles; 93 °C for 25 s, 60 °C for 35 s, and 72 °C for 20 s × 31 cycles. DNA sequence was read on ABI PRISM 3700 DNA Analyzer (Applied Biosystems). PCR efficiency was assessed according to the Ct value of FAM signal. BRAF V600E was regarded as positive when Ct value lowered than 28.

### Detection of the TERT mutations

TERT promoter C228T and C250T mutations were identified on genomic tumor DNA using standard PCR. Briefly, a 235-bp region of TERT promoter, containing the hotspots of C228T and C250T mutations, was PCR-amplified using primers 5′-AGTGGATTCGCGGGCACAGA-3′ (sense) and 5′-CAGCGCTGCCTGAAACTC-3′ (antisense). The thermal cycling conditions were as follows: 95 °C for 5 min × 1 cycle, 95 °C for 25 s, 64 °C for 20 s, and 72 °C for 20 s × 15 cycles; 93 °C for 25 s, 60 °C for 35 s, and 72 °C for 20 s × 31 cycles. After quality confirmation by agarose gel electrophoresis, PCR products were subjected to Sanger sequencing using ABI3500xl Dx Genetic Analyzer (Thermo Fisher, USA). When mutation was identified, an independent PCR amplification/sequencing, both in forward and reverse directions, was performed to confirm the result.

### Detection of NRAS mutation by real-time PCR

When genomic DNA isolation was finished, the detection of NRAS mutation in exon 2~4 was performed by AmoyDx® NRAS Mutation Detection Kit (Amoy Diagnostics, Xiamen, China). DNA (5 μL) was added to 35 μL PCR master mix, which contained PCR primers, fluorescent probes, PCR buffer, and DNA polymerase. The PCR cycling conditions were: 5 min denaturation at 95 °C, followed by 15 cycles of 95 °C for 25 s, 64 °C for 20 s, 72 °C for 20 s, 31 cycles of 93 °C for 25 s, 60 °C for 35 s, and 72 °C for 20 s. The PCR experiment was performed on ABI 7500 real-time instrument (Life Technologies, Carlsbad, CA, USA). Fluorescent signal was collected from FAM and HEX channels. NRAS mutation assay was determined according to the FAM Ct value.

### Statistical analysis

Quantitative data were expressed as means (±SD) for normally distributed variables or as medians and percentiles for non-normally distributed variables. The *t*-test was applied for variables that were normally distributed. Categorical variables were compared using *χ*^*2*^ tests. All *P* values were 2-sided and *P* less than 0.05 was considered significant. Analyses were performed using SPSS version 22.0 (SPSS Inc., Chicago, IL) and GraphPad Prism version 5 (GraphPad Software Inc., San Diego, USA). Review Manager 5.3 (Cochrane Collaborative, Oxford, UK) was used for meta-analysis.

## Results

### BRAF V600E gene mutation alone

**Fig. 1:**
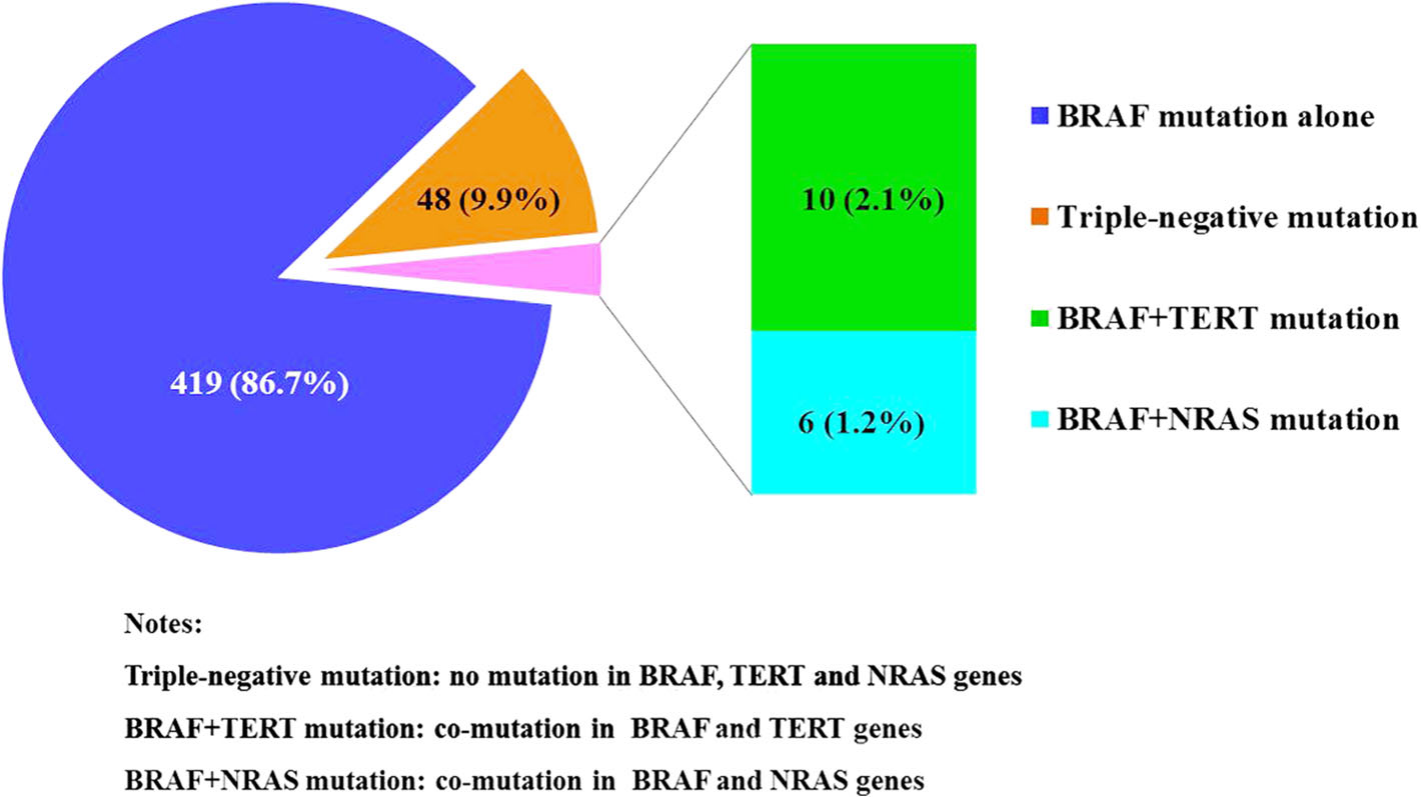
Distribution of BRAF V600E, TERT, NRAS mutations

As shown in Fig. 1, 435 (90.1%) patients with PTC harbored BRAF V600E mutation, including 419 patients with BRAF V600E mutation alone and 16 patients with double mutations. Interestingly, TERT and NRAS mutations were likely to coexist with BRAF V600E mutation in PTC. BRAF V600E and NRAS promoter double mutations were identified in 6 patients, with a prevalence of 1.2%. The mutant site of NRAS gene referred Exon2 G12D/G12S, Exon2 G12X/G13X and Exon3 Q61X. The average Ct value of NRAS gene was 24.23 ± 1.379. Meanwhile, we identified 10 (2.1%) cases of patients with TERT and BRAF V600E co-mutations, the most common mutant site of which was TERT C228T.

### Combined mutation of BRAF V600E with TERT or NRAS

Here we screened 16 (3.3%) patients with double mutations. Among them, 14/16 (87.5%) were female. The average age and BMI were 56.0 ± 11.0 and 25.38 ± 2.06, respectively. Other clinical characteristics were listed in Table 2. It seems like that PTCs with concurrent promoter mutations were associated with increased tumor aggressiveness. A majority of patients with double mutations possessed multiple lesions, metastatic lymph nodes, and achieved total thyroidectomy surgery.

**Table 2:** Clinical features and treatment patterns of 11 patients double mutations

No	Sex	Age	Blood type	BMI	Surgery	Operation time (min)	Invasion	Lesion location	Lesion number	Pathology	Stage	LNM	LNM location	Gene mutation	^131^I
1	F	29	O	27.5	a	100	Yes	Bilateral	Multiple	PTC + HT	mpT1bN1	1/1	lateral cervical LN	BRAF p.V600E(c.1800 T > A) (Ct = 18.31), NRAS Exon2 G12D/G12S (Ct = 21.88)	Yes
2	F	62	AB	24.4	a	60	Yes	Bilateral	Multiple	PTMC	T1a(3)N1a	1/4	Central LN	BRAF p.V600E (Ct = 19.46), NRAS Exon2 G12D/G12S (Ct = 24.5)	Yes
3	F	69	O	24.6	a	60	Yes	Bilateral	Multiple	PTC	T1b(m)N1b	11/15	Prelaryngeal and lateral cervical LN	BRAF p.V600E(c.1799 T > A) (Ct = 19.07), NRAS Exon3 Q61X (Ct = 26.57)	Yes
4	F	54	B	24.8	a	120	No	Bilateral	Multiple	PTC	T1bNx	1/2	Central LN	BRAF p.V600E) (Ct = 18.34), NRAS Exon3 Q61X (Ct = 24.03)	Yes
5	M	56	B	27.5	a	80	Yes	Unilateral	Single	PTC + HT	T1aN0	No	No	BRAF p.V600E(c.1800 T > A) (Ct = 18.03), TERT C228T	Yes
6	F	42	B	22.9	a	70	Yes	Unilateral	Single	PTC	T1bN1a	1/4	Central LN	BRAF p.V600E(Ct = 17.12), TERT C228T	Yes
7	F	66	A	27.1	a	180	Yes	Unilateral	Multiple	PTC	T3b(2) N1	1/12	lateral cervical LN	BRAF p.V600E(Ct = 19.11), TERT C228T,C250T	Yes
8	F	56	AB	23.0	b	85	No	Unilateral	Single	PTMC	T1aN1a	1/2	Central LN	BRAF p.V600E(Ct = 17.84), TERT C228T	No
9	F	62	B	23.4	a	160	Yes	Bilateral	Multiple	PTMC	T1a(2)N1	6/24	Central and lateral cervical LN	BRAF p.V600E(Ct = 18.7), TERT C228T	Yes
10	F	46	B	23.9	a	150	Yes	Unilateral	Multiple	PTC	T2 N1	7/16	Central and lateral cervical LN	BRAF p.V600E(Ct = 18.45), TERT C228T	Yes
11	F	58	A	28.8	a	60	No	Bilateral	Multiple	PTC	T1b(2)Nx	No	No	BRAF p.V600E(Ct = 18.72), TERT C228T	Yes
12	F	60	A	24.17	a	105	No	Unilateral	Single	PTMC	T1aN0	No	No	BRAF p.V600E (Ct = 17.7), NRAS Exon2 G12X/G13X (Ct = 23.8)	No
13	F	56	B	25.08	a	160	Yes	Unilateral	Multiple	PTC	T1bN0	No	No	BRAF p.V600E(Ct = 19.78), TERT C228T	No
14	F	43	A	27.05	b	75	No	Unilateral	Single	PTMC	T1aN0	No	No	BRAF p.V600E (Ct = 19.73), NRAS Exon3 Q61X (Ct = 24.57)	No
15	M	72	B	22.84	a	155	Yes	Unilateral	Single	PTC	T2 N1b	4/4	lateral cervical LN	BRAF p.V600E(Ct = 17.51), TERT C228T	Yes
16	F	67	O	29.07	b	80	No	Unilateral	Single	PTMC	T1aN0	No	No	BRAF p.V600E(Ct = 20.47), TERT C228T	No

*a* total thyroidectomy, *b* near-total thyroidectomy*, PTC* Papillary thyroid cancer, *PTMC* Papillary thyroid microcarcinoma, *HT* Hashimoto thyroiditis, *LNM* Lymph node metastasis

Compared the thyroid function before and after surgery (2.45 ± 1.2 months) of these 16 patients, the TSH [3.03 ± 1.65 (uIU/mL) vs 19.77 ± 39.7 (uIU/mL), *F* = 17.328, *P* < 0.01], T4 [109.86 ± 12.45(nmol/L) vs 108.98 ± 53.94 (nmol/L), *F* = 9.410, *P* = 0.005], FT4 [16.77 ± 2.05 (pmol/L) vs 20.01 ± 9.26 (pmol/L), *F* = 11.389, *P* = 0.003], FT3 [4.63 ± 0.51 (pmol/L) vs 4.28 ± 1.68 (pmol/L), *F* = 8.108, *P* = 0.009], Tg value [68.37 ± 137.06 (ng/mL) vs 1.25 ± 2.50 (ng/mL), *F* = 7.921, *P* = 0.01] were significantly different. The PTH [61.88 ± 31.5 (pg/mL) vs 48.8 ± 38.7 (pg/mL), *F* = 0.099, *P* = 0.76], T3[1.97 ± 0.23 (nmol/L) vs 1.51 ± 0.61 (nmol/L), *F* = 3.432, *P* = 0.076], TPO [54.01 ± 78.59 (U/mL) vs 54.08 ± 61.16 (U/mL), *F* = 0.129, *P* = 0.722], Atg [719.96 ± 1175.4(U/mL) vs 521.70 ± 778.51 (U/mL), *F* = 0.692, *P* = 0.414] remained relatively stable. So far, no recurrence, metastasis and mortality were observed.

### Relationship of gene mutations with clinicopathological outcomes of PTC

The risk factors for different gene mutations were explored. As Table 3 indicated, age (*F* = 16.704, *P* < 0.001), pathology (*χ*^*2*^ = 6.207, *P* = 0.045), number of lesions (*χ*^*2*^ = 7.169, *P* = 0.028), location of lesion (*χ*^*2*^ = 8.988, *P* = 0.011), lymph node metastasis (*χ*^*2*^ = 9.983, *P* = 0.007), and radiotherapy achievement (*χ*^*2*^ = 7.463, *P* = 0.024) were significantly different between 3 groups.

**Table 3:** Relationship between gene mutations and clinicopathologic features of PTC

	No gene mutation (*N* = 48)	BRAF V600E mutation alone(*N* = 419)	Double mutations (*N* = 16)	*χ* ^*2*^ */F*	*P*
Sex					
Male	13 (27.1%)	112 (26.7%)	2 (12.5%)	1.627	0.443
Female	35 (72.9%)	307 (73.3%)	14 (87.5%)		
Average age	37.9 ± 12.6	43.2 ± 10.7	56.0 ± 11.0	16.704	< 0.001
Average BMI	22.8 ± 3.57	24.9 ± 5.08	25.38 ± 2.06	3.521	0.316
Pathology					
PTC	23 (47.9%)	154 (36.8%)	10 (62.5%)	6.207	0.045
PTMC	25 (52.1%)	265 (63.2%)	6(37.5%)		
Lesion number					
Single lesion	38 (79.2%)	293(69.9%)	7 (43.8%)	7.169	0.028
Multiple lesions	10 (20.8%)	126(30.1%)	9 (56.2%)		
Lesion location					
Unilateral	38 (79.2%)	353 (84.2%)	9 (56.2%)	8.988	0.011
Bilateral	10 (20.8%)	66 (15.8%)	7(43.8%)		
Surgery					
Total thyroidectomy	39 (81.3%)	332 (79.2%)	13 (81.3%)	0.138	0.933
Near-total thyroidectomy	9 (18.7%)	87(20.8%)	3 (18.7%)		
LNM					
Yes	31 (64.6%)	180 (43.0%)	10 (62.5%)	9.983	0.007
No	17 (35.4%)	239 (57.0%)	6(37.5%)		
^131^I radiotherapy					
Yes	24 (50.0%)	163(38.9%)	11 (68.8%)	7.463	0.024
No	24 (50.0%)	256 (61.1%)	5 (31.2%)		

Multiple logistic regression analyses demonstrated that age [odds ratio (OR) = 1.044, 95% *confidence interval (CI)* = 1.013–1.076; *P* = 0.006], lymph node metastasis [OR = 0.094, 95% *CI* = 0.034–0.264; *P* < 0.001], and ^131^I radiotherapy [OR = 7.628, 95% *CI* = 2.721–21.378; *P* < 0.001] were significantly different between patients with or without BRAF V600E mutation (Table 4). For double mutant group, age [OR = 1.135, 95% *CI* = 1.069–1.205; *P* < 0.001], number of lesion (multiple/single) [OR = 4.128, 95% *CI* = 1.026–16.614; *P* = 0.046], and pathology (PTC/PTMC) [OR = 3.954, 95% *CI* = 1.235–12.654; *P* = 0.021] were independent factors (Table 5).

**Table 4:** Logistic regression analyses between BRAF V600E mutation group and BRAF V600E wild group

Index	*β*	SE	Wals	Sig.	HR	95%*CI*	
						upper	lower
Lymph node metastasis	−2.363	0.526	20.154	< 0.001	0.094	0.034	0.264
^131^I radiotherapy	2.032	0.526	14.930	< 0.001	7.628	2.721	21.378
Pathology (PTC/PTMC)	−0.418	0.334	1.564	0.211	0.659	0.342	1.267

**Table 5:** Logistic regression analyses between BRAF V600E mutation alone and double mutant group

Index	*β*	SE	Wals	Sig.	HR	95%*CI*	
						upper	lower
Age	0.126	0.031	17.008	< 0.001	1.135	1.069	1.205
Location of leision (bilateral/unilateral)	0.616	0.710	0.754	0.385	1.852	0.461	7.443
Number of leision (multiple/single)	1.418	0.710	3.983	0.046	4.128	1.026	16.614
Lymph node metastasis	−0.711	1.427	0.248	0.619	0.491	0.030	8.058
^131^I radiotherapy	0.840	1.487	0.319	0.572	2.315	0.126	42.70
Pathology (PTC/PTMC)	1.375	0.594	5.365	0.021	3.954	1.235	12.654

### Literature review of co-existence of BRAF V600E and TERT promoter mutations

**Fig. 2:**
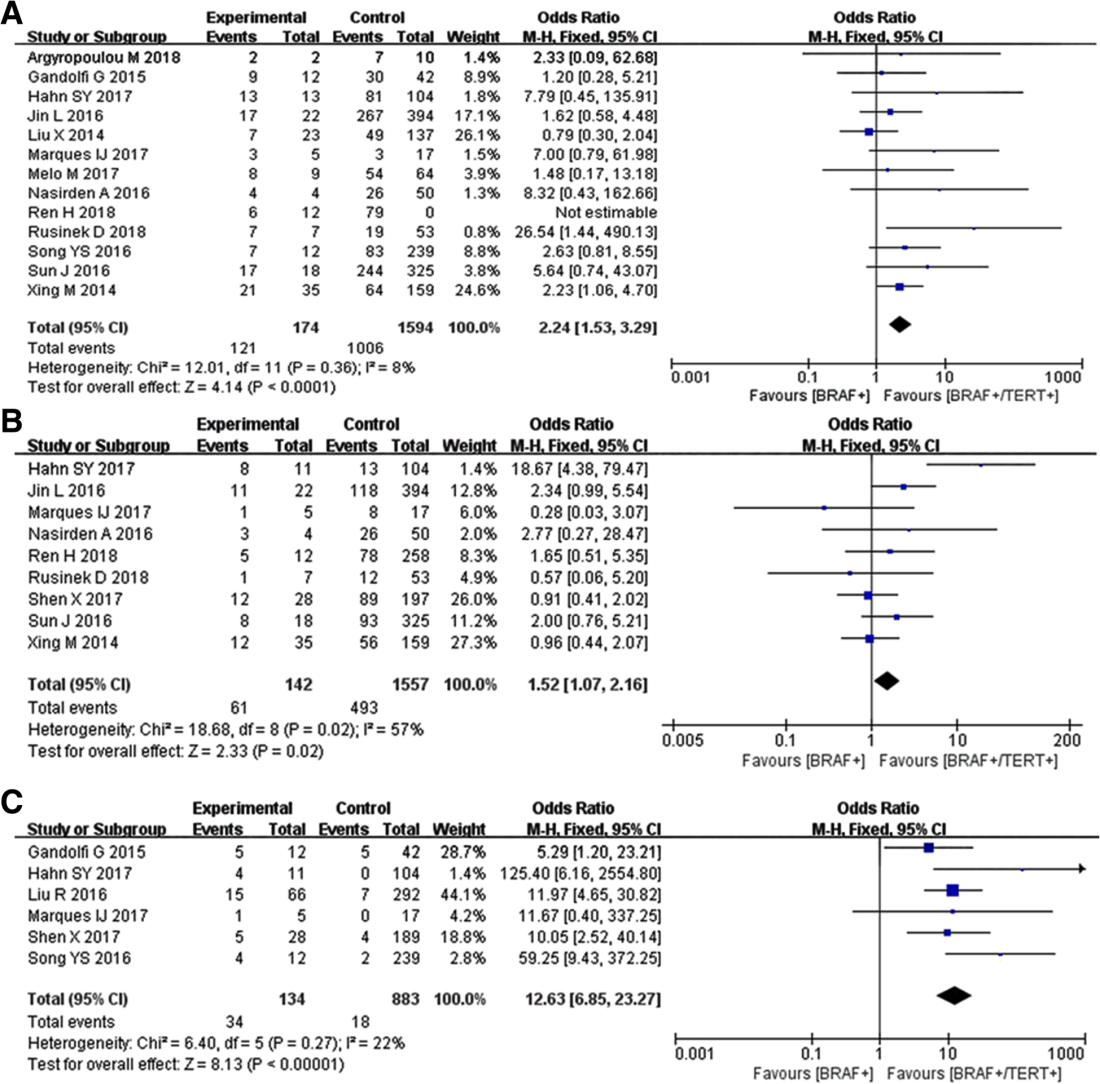
Systematic analysis of the association of BRAF promoter mutation alone or BRAF/TERT coexistence with clinicopathological features in thyroid cancer. **a** Lymph node metastasis, **b** Multifocality, **c** Dead of disease

**Fig. 3:**
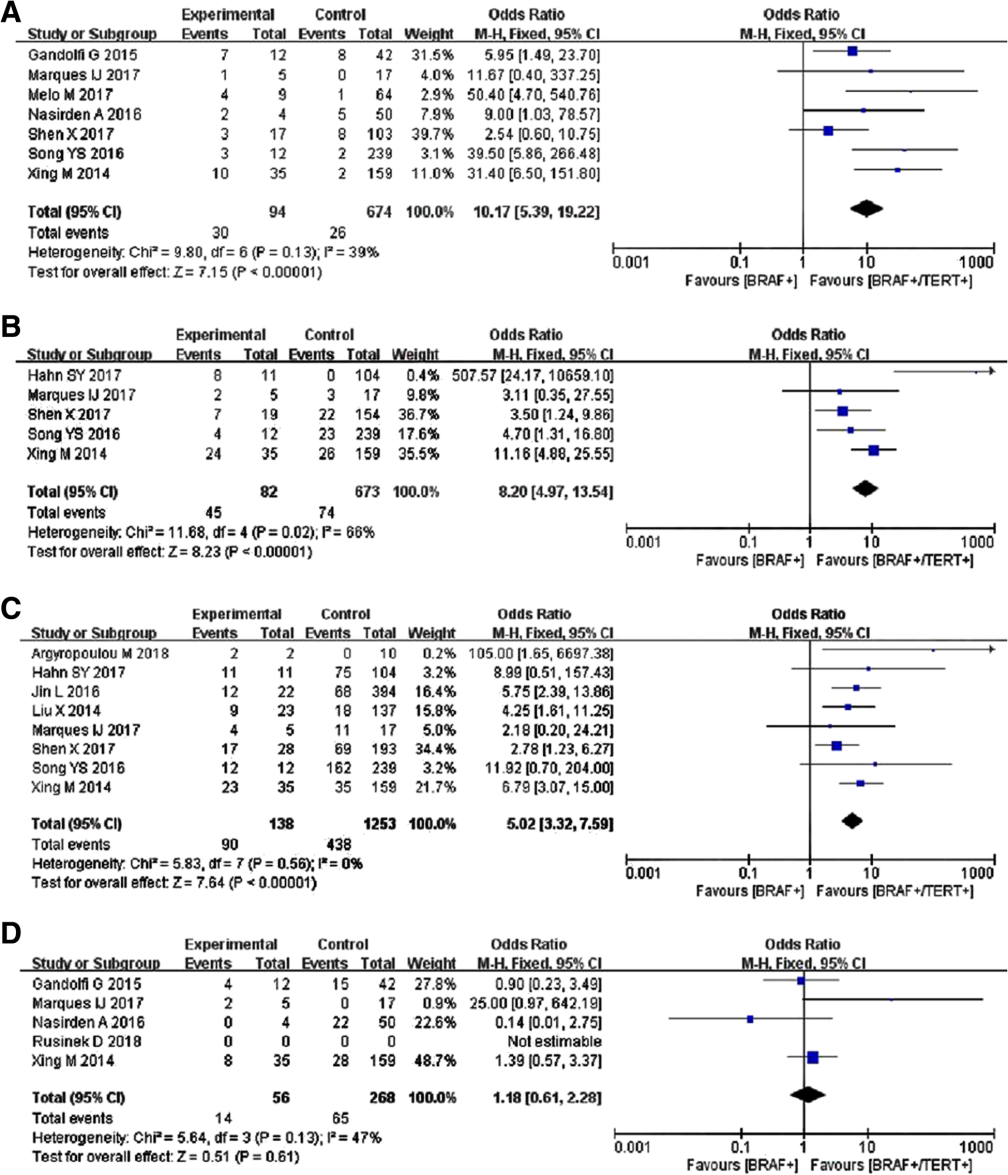
**a** Meta-analysis results of the relationship between BRAF V600E/TERT promoter mutations and distant metastasis. **b** Meta-analysis results of the relationship between BRAF V600E/TERT promoter mutations and tumor recurrence. **c** Meta-analysis results of the relationship between BRAF V600E/TERT promoter mutations and extrathyroidal extension. **d** Meta-analysis results of the relationship between BRAF V600E/TERT promoter mutations and vascular invasion

Systematic review was conducted to explore the impact of double gene mutations on clinicopathological features. Fifteen studies with 5057 participants, from inception to October 2018 were included [9, 12, 14–26]. Statistically meaningful association was found between BRAF V600E /TERT promoter co-mutations and lymph node metastasis (OR = 2.24, 95%*CI* = 1.53–3.29, *P* < 0.01, *I*^*2*^ = 8%, Fig. 2a), multifocality (OR = 1.52, 95%*CI* = 1.07–2.16, *P* = 0.02, *I*^*2*^ = 57%, Fig. 2b), dead of disease (OR = 12.63, 95%*CI* = 6.85–23.27, *P* < 0.01, *I*^*2*^ = 22%, Fig. 2c), distant metastasis (OR = 10.17, 95%*CI* = 5.39–19.22, *P* < 0.01, *I*^*2*^ = 39%, Fig. 3a), tumor recurrence (OR = 8.20, 95%*CI* = 4.97–13.54, *P* < 0.01, *I*^*2*^ = 66%, Fig. 3b), and extrathyroidal extension (OR = 5.02, 95%*CI* = 3.32–7.59, *P* < 0.01, *I*^*2*^ = 0%, Fig. 3c). Vascular invasion (OR = 1.18, 95%*CI* = 0.61–2.28, *P* = 0.61, *I*^*2*^ = 47%, Fig. 3d) was found without relationship with mutation coexistence.

## Discussion

In recent decades, the incidence of thyroid cancer has increased significantly, raising an imperative need to explore its pathogenesis, diagnosis, and treatment [27]. Genetic abnormalities maybe crucial in the tumorigenesis of thyroid cancer. Many molecule therapeutics, such as BRAF, has already undergone clinical trials, indicating the need to discover other markers for diagnosis and treatment prediction [28]. So far, the coexistence of gene mutation was focused. In 2014, Xing MZ et al. claimed firstly that the coexisting of BRAF V600E and *TERT* C228T mutations present the worst clinicopathologic outcomes [26]. Therefore, exploring the function of genetic events as prognostic markers for risk stratification and patient management is essential.

BRAF V600E mutation is the most frequent molecular alteration detected in PTC. But the mutation rate varies around the world. In 2015, Yip L et al. found the most common mutations were BRAF V600E (644/1039, 62%) in thyroid cancer patients from USA [29]. Identically, 62% BRAF V600E mutation was detected in Australia [30]. For Argentinean, 77% of patients operated for PTC harbored BRAF V600E mutation [31]. In 2017, Lee SE et al. reported the BRAF V600E mutation rate in Korean PTC patients was 80.8% [32]. Presently, the prevalence of BRAF V600E mutation of PTC patients was up to 88.2%, even higher than that in Korea. Hence, it is of great significance to obtain more evidence-based support of gene mutation in PTC patients.

Several studies have reported the coexistence of BRAF V600E and TERT gene mutations. However, it is still unclear why TERT promoter mutations most likely occur in cooperation with BRAF V600E mutation. In 2018, Ren H et al. found 3.5% PTC patients with co-existence of BRAF V600E and TERT promoter mutations [22]. In 2019, Colombo C et al. demonstrated that the double mutation rate of BRAF V600E and TERT promoter in aggressive PTC was 12% [33]. In this study, we observed 2.1% patients with BRAF V600E and TERT double mutations, lower than reported data around the world. Importantly, conflicting results were reported involving the clinical effects of BRAF V600E/TERT coexistence. In 2018, Jin A thought that patients with combined mutations were more likely to have a poor prognosis and outcome [11]. On the contrary, Nasirden A et al. found TERT/BRAF V600E double mutant tumors showed lower disease-free survival rate than BRAF V600E mutant tumors [21]. Presently, our meta-analysis provided strong evidence that BRAF V600E/TERT promoter mutations were significantly correlated with lymph node metastasis, multifocality, distant metastasis, tumor recurrence, extrathyroidal extension, and dead of disease. The meta-analysis by Vuong HG et al. achieved the same results. The combination of BRAF V600E and TERT promoter mutations could classify PTCs into four distinct risk groups with decreasing aggressiveness as follows: coexisting BRAF V600E and TERT > BRAF V600E alone > no mutations [13].

There are limited studies about NRAS gene mutation in PTC, still less about BRAF V600E and NRAS gene co-mutation. In 2017, Melo M et al. reported 1.2% mutation frequency of NRAS in primary PTCs [20]. Tobiás B et al. found 3.1% NRAS mutation in Hungarian Patients with PTC [34]. In 2018, NRAS promoter mutations were identified in 2 PTC cases, with a prevalence of 3.4% in the Greek Population [9]. In this study, the prevalence of NRAS mutation was 1.2%. NRAS promoter mutation was also likely to coexist with BRAF V600E mutation in PTC. However, the limited number of NRAS mutation interfered the research of its clinicopathological relationship. Because of the small number of NRAS mutation, we could not perform the clinicopathological relationship analysis. With the enlargement of mutant participants, we could obtain more promising evidence in the near future.

In conclusion, prevalence of BRAF V600E mutation in Northwest China was higher than other areas. Age, lymph node metastasis, and ^131^I radiotherapy were risk factors for BRAF V600E mutation. Age, multiple lesions, and pathology were independent factors for combination mutations. Coexistence of BRAF V600E and TERT promoter mutations were significantly correlated with lymph node metastasis, multifocality, distant metastasis, tumor recurrence, extrathyroidal extension, and dead of disease. The predictive value of NRAS combinational mutation with BRAF V600E needs more evidence.

## Data Availability

Yes

## Abbreviations

FFPE: Formalin-fixed and paraffin-embedded
PCR: Polymerase chain reaction
PTC: Papillary thyroid cancer
TERT: Telomerase reverse transcriptase
